# The Human PNPLA3^I148M^ Polymorphism Alters the Renal Kinome and Transporters to Cause Hyperfiltration and Hypertension

**DOI:** 10.1096/fba.2026-00222

**Published:** 2026-09-19

**Authors:** Gertrude Arthur, Michael I. Adenawoola, Sally Wahba, Bentley S. Montgomery, Claire B. Wingfield, Zachary A. Kipp, Terry D. Hinds, David E. Stec

**Affiliations:** ^1^ Department of Physiology & Biophysics, Cardiorenal, and Metabolic Diseases Research Center University of Mississippi Medical Center Jackson Mississippi USA; ^2^ Department of Pharmacology and Nutritional Sciences University of Kentucky Lexington Kentucky USA; ^3^ Barnstable Brown Diabetes Center University of Kentucky Lexington Kentucky USA

**Keywords:** hyperfiltration, hypertension, kidney, kinome, liver, transporters

## Abstract

The patatin‐like phospholipase domain‐containing protein 3 (Pnpla3) I148M polymorphism is a well‐established genetic determinant of metabolic dysfunction‐associated steatotic liver disease (MASLD), and emerging evidence links it to chronic kidney disease (CKD) and cardiovascular disease (CVD). However, the mechanisms underlying this relationship remain unclear. The goal of this study was to investigate the role of the Pnpla3^I148M^ polymorphism on kidney function, blood pressure, and cardiac function in a mouse model carrying the identical human polymorphism. Male and female Pnpla3^I148M^ knock‐in and control C57BL/6 mice were fed either a normal sucrose diet (NSD) or a high sucrose diet (HSD) for 8 weeks. Glomerular filtration rate (GFR), blood pressure, cardiac, and vascular function were measured in each group. Renal cortical protein expression and kinome profiles were analyzed to identify pathways associated with hyperfiltration and renal injury. Pnpla3^I148M^ mice exhibited increased renal sodium transporter levels and hyperfiltration, which was compounded by albuminuria in HSD mice. Pnpla3^I148M^ mice were also hypertensive and exhibited impaired systolic function, consistent with pressure‐overload‐induced cardiac remodeling. Renal kinome profiling revealed sex‐ and diet‐dependent alterations in serine/threonine kinases, primarily involving isoforms of protein kinase C (PKC), protein kinase A (PKA), cGMP‐dependent protein kinase (PRKG), and calcium/calmodulin‐dependent protein kinase (CaMK). Our findings suggest that Pnpla3^I148M^ acts as a systemic cardio‐renal risk allele that links metabolic dysregulation to progressive end‐organ damage via renal kinase remodeling in a sex‐ and diet‐dependent manner.

## Graphical abstract

The present study used mice carrying the I148M polymorphism in the human Patatin‐like phospholipase domain‐containing protein 3 (PNPLA3^I148M^) to determine the role of this polymorphism in regulating blood pressure and renal filtration. Kinome analysis revealed that male PNPLA3^I148M^ mice exhibited decreased phosphorylation of protein kinase c alpha (PKCα), resulting in increased levels of the sodium‐hydrogen exchanger 3 (NHE3), hypertension, and renal hyperfiltration. Female PNPLA3^I148M^ mice exhibited increased levels of phosphorylated calcium/calmodulin‐dependent protein kinase type IV (CamK4), activated levels of the Na/K‐ATPase, hypertension, and renal hyperfiltration. These results demonstrate sex‐specific changes in renal kinase activity that affect renal transporters, leading to renal hyperfiltration and hypertension. These results may explain the association between PNPLA3^I148M^ and the development of chronic kidney disease.

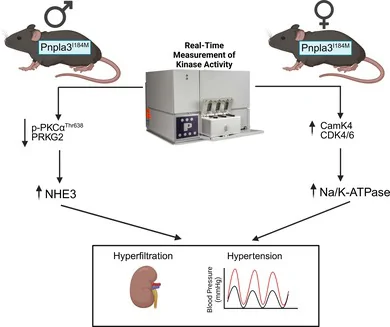

## 1 Introduction

Polymorphism in the patatin‐like phospholipid domain‐containing protein 3 (Pnpla3) is linked to metabolic dysfunction‐associated steatotic liver disease (MASLD) [1, 2]. MASLD is highly associated with metabolic diseases such as obesity, diabetes, and dyslipidemia. MASLD currently affects more than 40% of the US population [3]. A mutation in the Pnpla3 protein at amino acid residue 148, where methionine is substituted for isoleucine (Pnpla3^I148M^), has emerged as a genetic risk factor for the development of MASLD [4, 5]. This mutation leads to both loss‐ and gain‐of‐function effects on Pnpla3's lipolytic activity. As a loss of function, Pnpla3^I148M^ reduces the hydrolysis of polyunsaturated fatty acids in lipid droplets [6, 7]. As a gain‐of‐function, Pnpla3^I148M^ evades ubiquitination and surrounds lipid droplets, leading to lipid accumulation [4, 8, 9]. Hence, the Pnpla3^I148M^ polymorphism is a major driver of MASLD [4, 5]. The prevalence of the Pnpla3^I148M^ polymorphism is high (> 40%) in South America and Asia, with a moderate prevalence (20%–35%) in North America and Europe [10, 11, 12].

The primary cause of mortality in MASLD patients is cardiovascular disease. Cardiovascular diseases (CVDs) are the leading cause of death worldwide and account for about 40% of mortality in MASLD patients in the US [13]. However, population studies examining the association between the Pnpla3^I148M^ polymorphism and MASLD, as well as CVDs, have been inconclusive regarding its role in CVD mortality among MASLD patients.

One school of thought is that the Pnpla3^I148M^ polymorphism may actually protect against the development of CVD by lowering circulating triglyceride and lipoprotein levels. In this regard, recent studies have reported that the Pnpla3^I148M^ polymorphism is negatively associated with coronary artery disease [14], aortic stiffness [15], and the development of cardiovascular disease [16]. However, recent studies have shown that the relationship between genetically driven liver fat accumulation and coronary heart disease (CHD) risk may be mediated by ApoB‐containing lipoproteins [17]. Pnpla3^I148M^ has no effect on ApoB levels and promotes hepatic steatosis, both of which may neutralize any potential protective effects on circulating triglyceride and lipoprotein levels. Interestingly, a recent population study in the US, involving 4814 participants and a 20‐year median follow‐up, the Pnpla3^I148M^ polymorphism was associated with increased overall mortality in both MASLD and non‐MASLD participants in a dose‐dependent manner; increased CVD mortality, when adjusted for age and sex, and a trend for higher CVD mortality in multivariate analysis [18]. These results suggest that the Pnpla3^I148M^ polymorphism increases the risk of CVD mortality, independent of MASLD, through unknown mechanisms.

New evidence also suggests that the Pnpla3^I148M^ polymorphism increases the incidence of chronic kidney disease (CKD) and its progression in both pediatric and adult patients, independent of other comorbidities, such as type 2 diabetes and MASLD [19, 20, 21, 22]. CKD affects 1 in 7 adults in the US [23] and is predicted to become the 5th leading cause of death worldwide by 2040 [24]. CKD is also a significant risk factor for the development of CVD [25, 26]. Dialysis patients in the US have a 40% 5‐year survival rate, mainly due to cardiovascular complications that arise as CKD progresses [27]. Hence, CKD incidence may explain the higher mortality in people with the Pnpla3^I148M^ polymorphism. Yet, little is known about how the Pnpla3^I148M^ polymorphism leads to CKD.

The goal of this study was to investigate the role of Pnpla3^I148M^ polymorphism on kidney function, blood pressure regulation, and cardiac function using a mouse model of Pnpla3^148M^ knock‐in.

## 2 Methods

### 2.1 Animals

Mice with endogenous knock‐in of Pnpla3^I148M^ were obtained from Regeneron (Tarrytown, NY, USA). Homozygous Pnpla3^I148M^ mice have been bred on a C57BL/6 background for more than ten generations. C57BL/6 mice, used as controls, were obtained from Jackson Laboratories (Bar Harbor, ME, USA). All animal studies described below complied with the University of Mississippi Medical Center Institutional Animal Care and Use Committee (protocol no. 2022–1219) and followed the “Guide for the Care and Use of Laboratory Animals” issued by the National Institutes of Health. Mice were on a 12:12 h light:dark cycle, starting at 6:00 am for the light cycle and at 6:00 pm for the dark cycle, and had ad libitum access to food and water. Mice were fasted for 6 h from early morning to afternoon before euthanasia, and blood and tissues were collected and stored at −80°C for further analysis.

### 2.2 Experimental Design

Sixteen‐week‐old Pnpla3^I148M^ and C57BL/6 mice were kept on either a normal sucrose diet (3.1 kcal/g; Teklad Harlan, Madison, WI, USA) or randomized to a high sucrose diet (4.2 kcal/g, 68% sucrose by weight, MP Biosciences, Irvine, CA, USA) for 8 weeks (*n* = 10–16 mice/group). A high‐sucrose diet has been shown to induce hepatic steatosis in Pnpla3^I148M^ mice to a greater extent than in control mice [28]. Glomerular filtration rate (GFR) was assessed at Week 7. Mice were placed in metabolic cages (Techniplast Solo Mouse Metabolic Cages, Exton, PA, USA) at the end of Week 7 with free access to food and water for urine collection. Cardiovascular function and blood pressure were assessed at Week 8, and then mice were euthanized for tissue and plasma collection.

### 2.3 Measurement of Glomerular Filtration Rate

Transdermal GFR assessment was performed at Week 7 using Fluorescein isothiocyanate (FITC)‐Sinistrin (5 mg/100 g BW) as previously described [29]. Mice were lightly and briefly anesthetized to remove a small area of fur on the flank using an electric shaver, followed by application of depilatory cream. Mice with large dark patches on the flanks were excluded from measurements because the GFR probe cannot accurately record clearance in these mice. Using a double‐sided adhesive patch, the Medi‐Beacon transdermal device (MediBeacon Inc., St. Louis, MO, USA) was placed on a shaved area on the flank and secured with an adhesive tape. A baseline recording was performed for 15 min, followed by an injection of FITC‐Sinistrin via retro‐orbital injection. Mice were then left in individual cages for 2 h for clearance recording. FITC‐Sinistrin elimination kinetics was calculated using the Medi‐Beacon studio 3 software to determine the half‐life of Sinistrin (*t* ^1/2^). GFR was calculated as $\frac{\text{Conversion factor}\;\left(14616.8\right)}{\frac{t_{1}}{2}\left(\text{FITC}‐\text{Sinistrin}\right)\;\left(\text{minutes}\right)}$.

### 2.4 Measurement of Cardiovascular Function

Mouse transthoracic echocardiographic measurements were performed using the Vevo 3100 high‐resolution imaging system (FUJIFILM VisualSonics, Toronto, ON, Canada) as previously described [30, 31, 32]. Hearts were imaged with the ultra‐high‐frequency MX 400 probe: the parasternal long‐axis view in B‐mode and the short‐axis view in M‐mode. Recordings from B and M‐modes were used to evaluate systolic function. An apical four‐chamber B‐mode view was used to assess diastolic function by pulsed‐wave Doppler and tissue Doppler imaging. A longitudinal B‐mode view of the abdominal aorta (AA) and the left common carotid artery (LCCA) was used to assess vascular compliance by pulse‐wave Doppler imaging.

### 2.5 Measurement of Blood Pressure

Blood pressure was measured using indwelling fluid‐filled catheters implanted into the left carotid artery, as previously described (29). Mice were given buprenorphine‐SR as an analgesic prior to surgery (Ethiqa XR, Fidelis, North Brunswick, NJ, USA). Blood pressures were measured in 4‐h periods on two consecutive days in conscious, freely moving mice. Blood pressure data were continuously collected by a PowerLab 8/sp. polygraph (AD Instruments, Denver, Colorado, USA) and acquired on a computer running Chart 4 software provided by the manufacturer. Blood pressure data are presented as the average of the two‐day measurements.

### 2.6 Plasma, Urine, and Tissue Analysis

Plasma cystatin C (R&D Systems, San Jose, CA, USA, #MSCTC0), and urinary albumin (Ethos Biosciences, Philadelphia, PA, USA, #r10082024) were measured by ELISA according to the manufacturer's instructions. Urinary creatinine was measured using a VETAXEL chemistry analyzer (Alfa Wasserman, West Caldwell, NJ, USA). Colorimetric triglyceride assay was performed on frozen kidney cortex from NSD and HSD mice using a triglyceride kit (Abcam, Waltham, MA, USA, #Ab65336) according to the manufacturer's instructions.

### 2.7 Immunoblotting

Protein from frozen kidneys was extracted using gentleMACS Tissue Dissociator and Tubes (Miltenyi Biotec, Gaithersburg, MD, USA) with T‐PER Tissue Protein Extraction Reagent (Invitrogen‐Thermo Fisher Scientific, Waltham, MA, USA) and protease and phosphatase inhibitor cocktails. Protein levels were quantified using SmartSpec 3000 UV/Vis Spectrophotometer (Bio‐Rad Laboratories, Hercules, CA, USA) and 40 μg of protein was run on a precast polyacrylamide gel. Proteins were then transferred to a nitrocellulose membrane using a semi‐dry transfer system (Trans‐Blot Turbo Transfer System; Bio‐Rad Laboratories, Hercules, CA, USA) as recommended by the manufacturer. Membranes were blocked in Tris‐buffered saline with 0.1% Tween 20 for 1 h. Membranes were then incubated with NHE3 (StressMarq, British Columbia, Canada, #402), α‐ENaC (StressMarq, British Columbia, Canada, #403), Alpha 1 Na‐K‐ATPase (Cell signaling technology, Danvers, MA, USA, #3010), phosphor PKCα (Th638: Cell signaling technology, Danvers, MA, USA, #9375, Th497: Abcam, Waltham, MA, USA, #76016), total PKCα (Invitrogen‐Thermo Fisher Scientific, Waltham, MA, USA, #MA1‐157), phosphor GSK3β (Cell signaling technology, Danvers, MA, USA, #9323), total GSK3β (Cell signaling technology, Danvers, MA, USA, #9832), and GAPDH (Proteintech, Rosemont, IL, USA, #60004) in Tris‐buffered saline with 0.1% Tween 20. After incubation with FITC secondary antibodies, anti‐mouse and anti‐rabbit (LICOR, Lincoln, NE, USA, #926‐32211, 926‐68052), proteins were imaged using Chemidoc MP (Bio‐Rad Laboratories, Hercules, CA, USA). Protein levels were quantified using ImageLab software (Bio‐Rad Laboratories, Hercules, CA, USA).

### 2.8 Histology

A separate cohort of mice (*n* = 5–7/group) was kept on either an NSD or an HSD as described above. At 8 weeks of diet, mice were euthanized, and the heart and kidney were preserved in formalin and then paraffin‐embedded for sectioning and staining. Picrosirius red (PSR) was done for the heart, and periodic acid–Schiff (PAS) for the kidney. Histological images of PSR were captured at 4× and 10× magnification using the color‐brightfield camera on the BioTek Lionheart FX automated microscope (Agilent Technologies, Santa Clara, CA) and the Mantra 2 Quantitative Pathology Imaging microscope (Akoya Biosciences, Billerica, MA), respectively. Quantification was performed on 10X images using Image J. Measurements from 4 to 6 sections per individual animal were averaged, and the individual averages were used to obtain the group averages.

Images for PAS staining were taken at 20× magnification using the Mantra 2 Quantitative Pathology Imaging microscope (Akoya Biosciences, Billerica, MA). Quantification of kidney injury was done in a blinded, semiquantitative manner using an established scoring scale [33]. Percentages of glomeruli in slide section that displayed glomerulomegaly, mesangial expansion, fibrosis/sclerosis, parietal cell expansion, tubular cell injury, and inflammation were scored as follows: 0 = none, 1 = < 25%, 2 = 25%–50%, 3 = 50%–75%, and 4 = > 75%.

### 2.9 Renal Kinome Assessment

Serine–threonine kinases and protein‐tyrosine kinases (STK #3250, PTK #32508) were measured using the PamChip4 porous three‐dimensional (3D) microarrays and PamStation12 (PamGene International, 's‐Hertogenbosch, the Netherlands). Renal cortices from each group were pooled and measured in triplicate across three chips simultaneously as previously described [34, 35]. This approach minimizes large batch effects across samples. Male and female STK were measured separately, with diet comparisons (CTL NSD vs. Pnpla3^I148M^ NSD; CTL HSD vs. Pnpla3^I148M^ HSD). The pooled samples were lysed using M‐PER mammalian extraction buffer (Thermo Fisher Scientific, #78503), halt phosphatase inhibitor (Thermo Fisher Scientific, Waltham, MA, USA, #78428), and protease inhibitor cocktail (Sigma‐Aldrich St. Louis, MO, USA, #P2714). The samples were homogenized using gentleMACS Tissue Dissociators (Miltenyi Biotec, Gaithersburg, MD, USA). The protein concentration was measured in duplicate using Pierce BCA Protein assay (Thermo Fisher Scientific, Waltham, MA, USA, #23225). Samples were diluted to a final protein concentration of 5 μg/μL for each array. In the presence of ATP, kinase phosphorylation activity is quantified using fluorescently labeled antibodies to detect differential phosphorylation of 144 (STK) reporter peptides between groups. Evolve (PamGene) software uses a charge‐coupled device (CCD) camera and light‐emitting diode (LED) imaging system to record relative phosphorylation levels of each unique consensus phosphopeptide sequence every 5 min for 60 min. The images taken were analyzed using BioNavigator (PamGene, 's‐Hertogenbosch, the Netherlands). Signal ratios are used to calculate fold change (FC) for each phosphopeptide sequence averaged across three replicates. A median final score in differential phosphopeptide signal values greater than or equal to 30% (FC ≥ 1.30) was considered significant. Volcano plots were generated by BioNavigator (PamGene, 's‐Hertogenbosch, the Netherlands). Kinome phyla were generated using CORAL [36] by plotting the significant median final score (FC ≥ 1.30) and kinase statistic. Using the peacock plot analysis [34], we identified the differentially phosphorylated peptide substrates targeted by each serine/threonine kinase using a 2000‐random‐sampling iteration (KRSA). Measurements extensively of winners (MEOW) plots were used to quantify individual kinase activities on substrates, examining the confidence of the experimental versus the control groups using the equation: (log_2_ fold change (FC) of kinase substrates × ∆confidence [experimental hits/mean hits of 2000 random sampling iterations]).

### 2.10 Statistical Analysis

All data were analyzed with Prism 11 (GraphPad Software, San Diego, CA, USA) using two‐way ANOVA with Tukey's post hoc test to compare pairs of group means and identify interactions. *p* values of 0.05 or less were considered significant. Results are expressed as mean ± SEM.

## 3 Results

### 3.1 Pnpla3^I148M^ Polymorphism Leads to Renal Hyperfiltration and Injury

**FIGURE 1:**
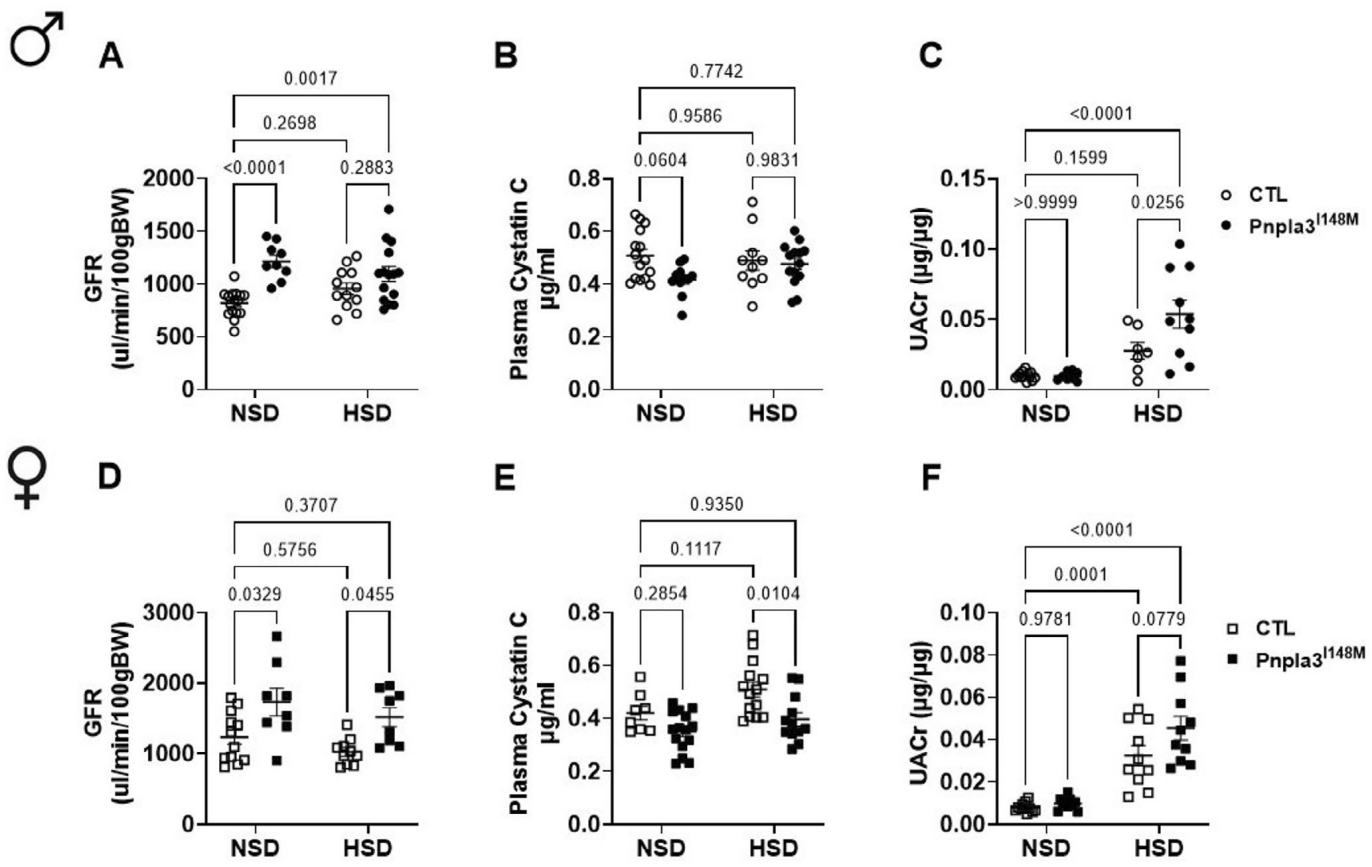
Pnpla3^I148M^ polymorphism leads to renal hyperfiltration and injury. Direct glomerular filtration rate (GFR) assessment by FITC‐Sinistrin in (A) males (D) females. Indirect GFR assessment by plasma cystatin C in (B) males (E) females. Urinary albumin to creatinine ratio (UACr) assessment in (C) males (F) females. Data are expressed as mean ± SEM of 10–15 mice/group. A two‐way ANOVA was performed to detect differences.

Direct glomerular filtration rate (GFR) assessment by transdermal tracing of FITC‐Sinistrin clearance showed increased GFR in Pnpla3^I148M^ mice, with the exception of male HSD mice (Figure 1A,D). HSD male Pnpla3^I148M^ mice showed increased GFR only when compared to NSD controls but not to matched HSD controls. Indirect GFR assessment by plasma cystatin C showed similar results as obtained with the direct FITC‐Sinistrin measurements, with an increase in GFR in female HSD Pnpla3^I148M^ mice, a trend for GFR increase in NSD Pnpla3^I148M^ mice, and no change in GFR in male HSD Pnpla3^I148M^ mice (Figure 1B,E). Urinary albumin to creatinine ratio (UACr) was increased only in male HSD Pnpla3^I148M^ mice, and female HSD mice had a trend for increased UACr (Figure 1C,F).

**FIGURE 2:**
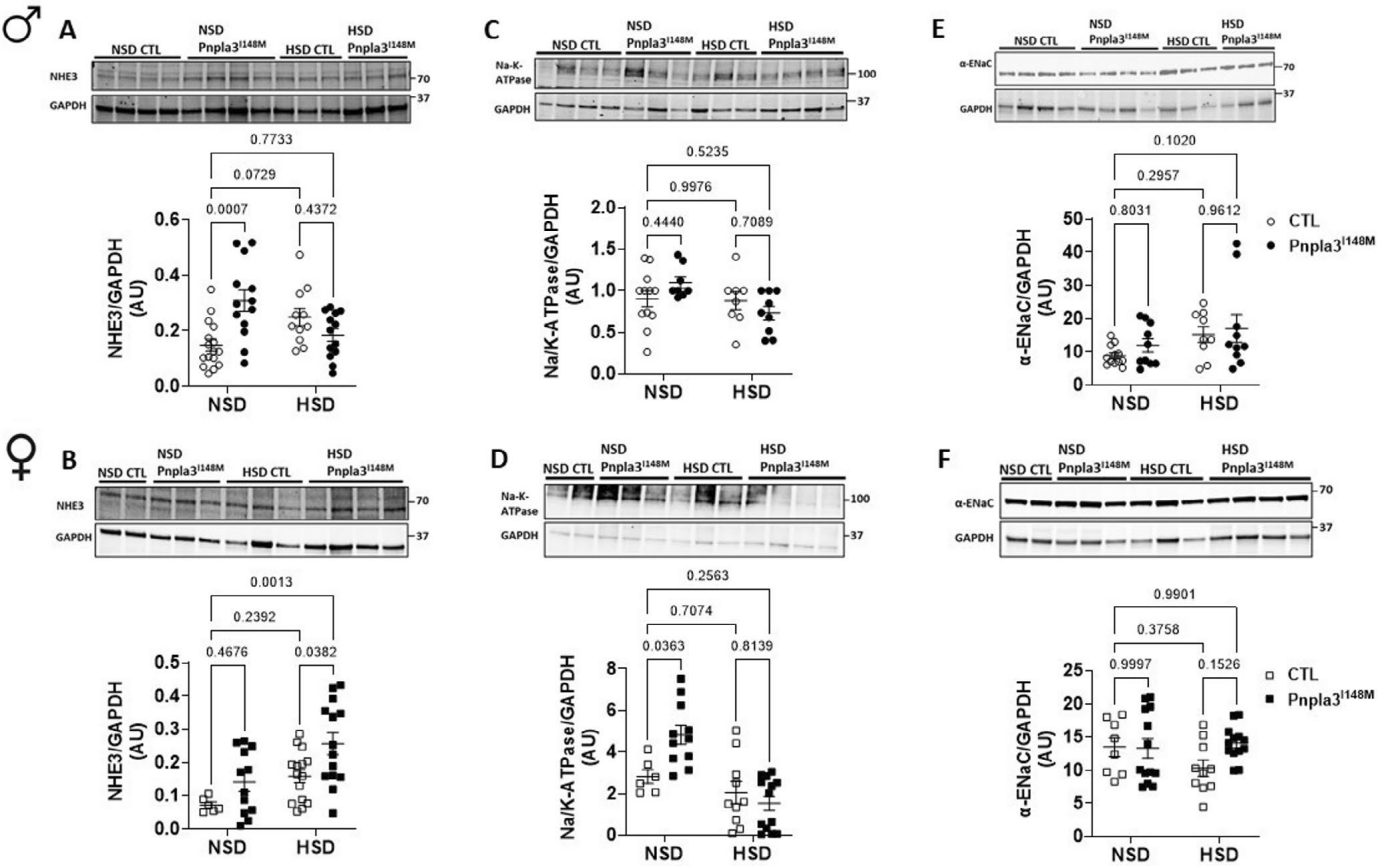
Hyperfiltration is due to increased expression of sodium transporters in renal cortex. Representative western blots and quantification of sodium‐hydrogen exchanger 3 (NHE3) in (A) males and (D) females. Representative western blots and quantification of sodium potassium ATPase (Na^+^/K^+^‐ATPase) in (B) males and (E) females. Representative western blots and quantification of the alpha subunit of epithelial sodium channel (α‐ENaC) in (C) males and (F) females. Data are expressed as mean ± SEM of 8–15 mice/group. A two‐way ANOVA was performed to detect differences.

To determine the cause of hyperfiltration, we assessed protein levels of sodium transporters in the renal cortex, namely, the sodium/hydrogen exchanger 3 (NHE3), the sodium‐potassium ATPase (Na^+^/K^+^‐ATPase), and the alpha subunit of the epithelial sodium channel (α‐ENaC). Western blot analysis showed an increase in NHE3 protein levels in NSD male and HSD female Pnpla3^I148M^ mice (Figure 2A,B). The Na^+^/K^+^‐ATPase protein levels were increased in only NSD females (Figure 2C,D). Protein levels of α‐ENaC were unchanged in all groups, but HSD Pnpla3^I148M^ mice showed a moderate trend for increased levels as compared to NSD controls. (Figure 2G,H).

### 3.2 Pnpla3^I148M^ Mice Show Alterations in Cardiac Function, Are Hypertensive, and Exhibit Sex‐Dependent Alterations in Vascular Function

**FIGURE 3:**
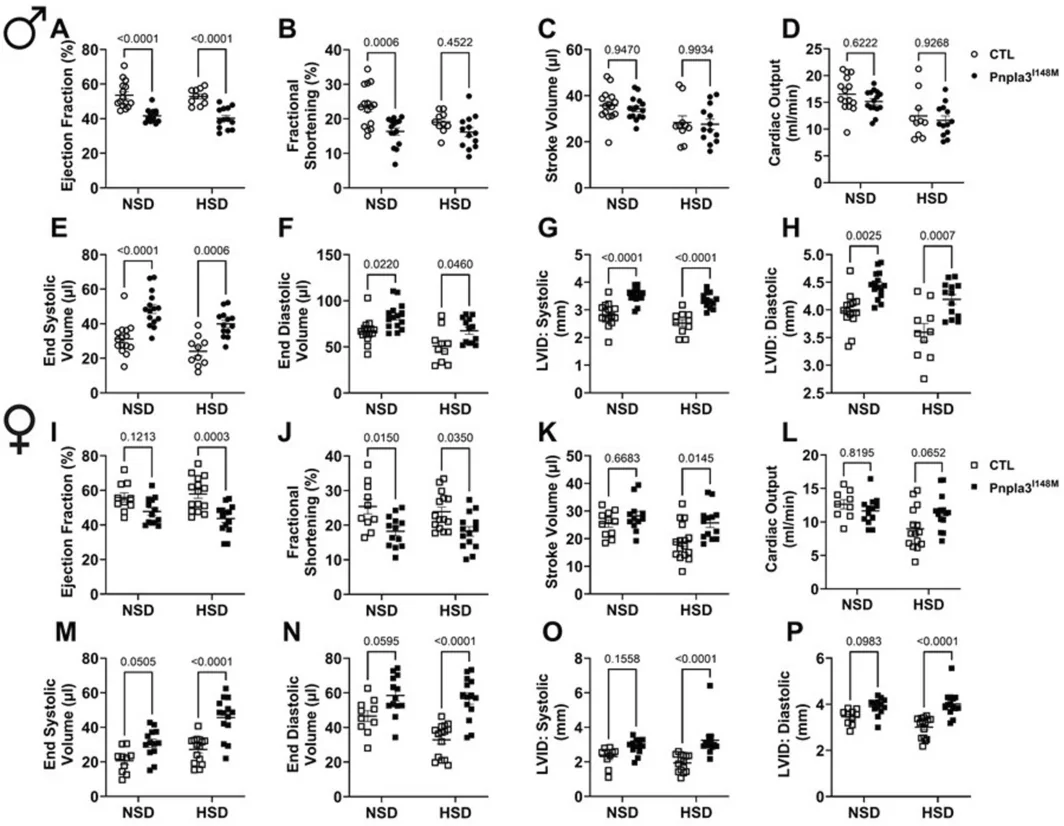
Pnpla3^I148M^ mice show alterations in cardiac function. Echocardiographic analysis of the heart in B and M mode was used to determine Ejection fraction in (A) males (I) females. Fractional shortening in (B) males (J) Females. Stroke volume in (C) males (K) females. Cardiac output in (D) males (L) Females. Data from B and M modes were averaged for each mouse and then for each group. Echocardiographic analysis of the heart in M mode was used to determine End systolic volume in (E) males (M) females. End diastolic volume in (F) males (N) females. Left ventricular internal dimension (LVID) at systole in (G) males (O) females. LVID at diastole in (H) males (P) females. Data are expressed as mean ± SEM of 10–15 mice/group. A two‐way ANOVA was performed to detect differences.

Echocardiographic assessment of the heart showed that Pnpla3^I148M^ polymorphism led to a reduction in ejection fraction and fractional shortening (Figure 3A,B,I,J) but did not change stroke volume or cardiac output (Figure 3C,D,K,L). Male Pnpla3^I148M^ mice also exhibited an increase in end‐systolic and diastolic volume, left ventricular internal diameter (LVID) at systole and diastole, and mitral valve early filling (MV E), irrespective of diet (Figure 3E–H, Table S1). In females, end‐systolic and diastolic volume, as well as LVID at systole and diastole, and area of heart at systole and diastole, were increased only in HSD Pnpla3^I148M^ mice (Figure 3M–P, Table S2). Left ventricular anterior wall (LVAW) at systole was only increased in NSD Pnpla3^I148M^ mice (Tables S1 and S2).

**FIGURE 4:**
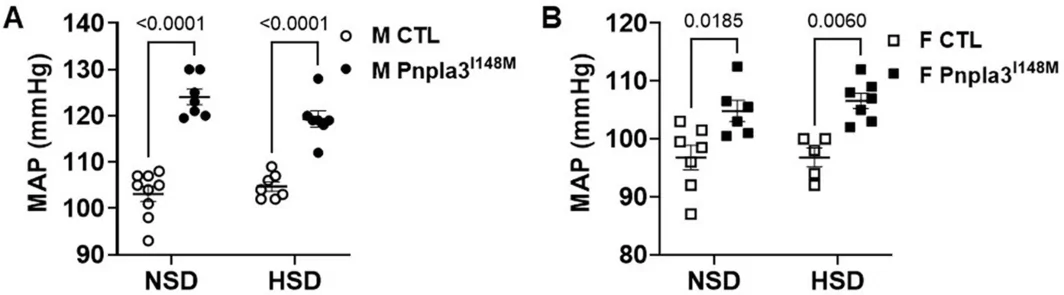
Pnpla3^I148M^ is hypertensive irrespective of diet. Blood pressure measured in (A) males and (B) females. Blood pressure was measured by fluid‐filled catheter in conscious, unrestrained mice for 4 h on two consecutive days and then averaged. Data are expressed as mean ± SEM of 5–9 mice/group. A two‐way ANOVA was performed to detect differences.

Blood pressure, measured in conscious, freely moving mice, was elevated in female and markedly increased in male Pnpla3^I148M^ mice compared to controls, irrespective of diet (Figure 4A,B). No changes were observed in vascular structure and function in male Pnpla3^I148M^ mice, irrespective of diet (Figure S1A–F, Table S1), but vascular compliance in females was decreased. Female Pnpla3^I148M^ mice also exhibited increased abdominal aorta pulsatility and resistive index, irrespective of diet (Figure S1G,H), and HSD females also showed increased left common carotid pulsatility and resistive index (Figure S1K,L). There were no changes in the peak systolic, mean velocity, and mean gradient of the abdominal aorta (Figure S1I,J, Table S2). There was no change in cardiac collagen deposition, as measured by PSR staining in Pnpla3^I148M^ mice compared to controls (Figure S2A–D).

### 3.3 Pnpla3^I148M^ Polymorphism Increases Renal Lipid Storage and Injury in HSD Male Mice

**FIGURE 5:**
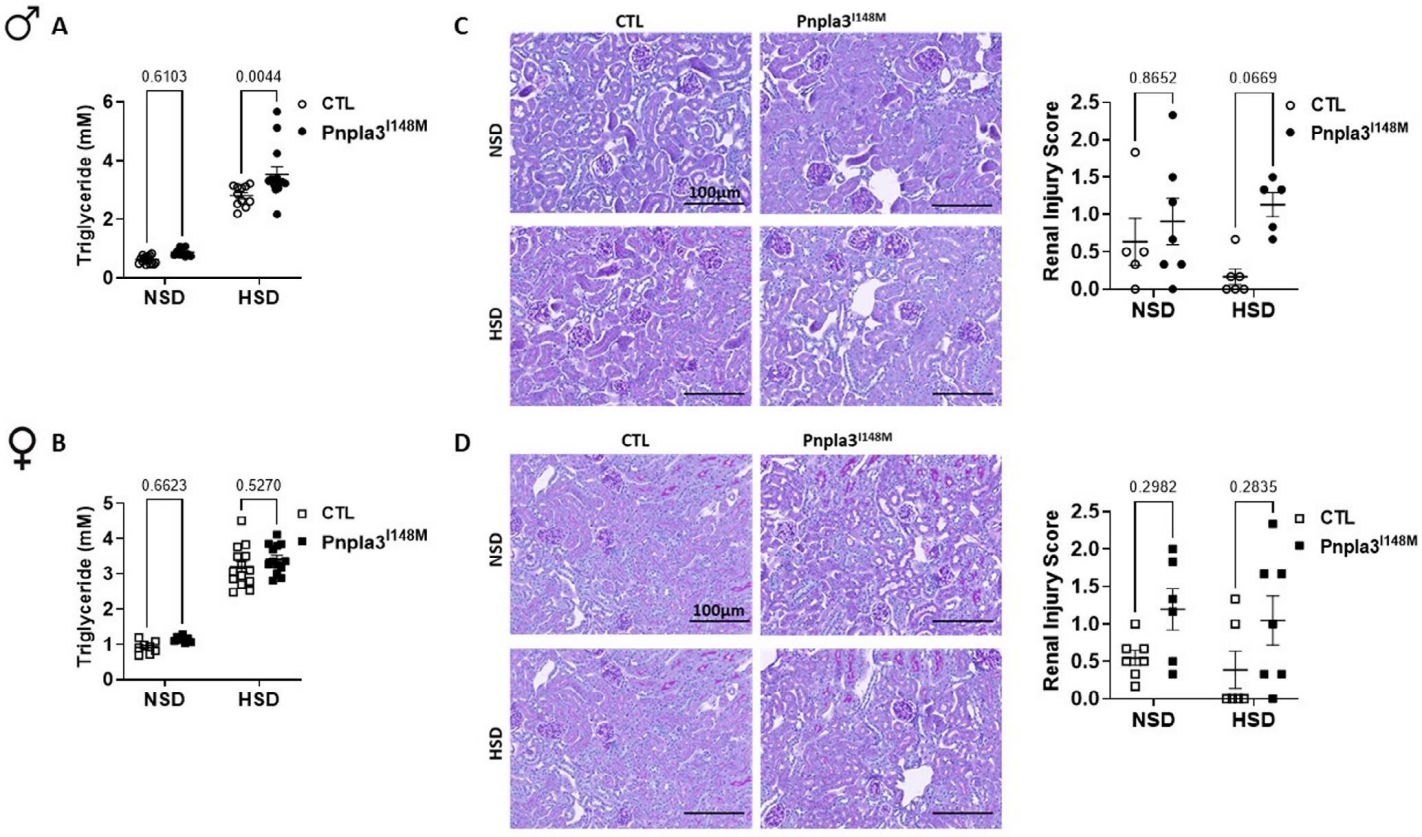
HSD Pnpla3^I148M^ mice have increased renal lipid storage and injury. Renal cortical triglyceride levels in (A) male and (C) female mice measured by ELISA. Representative image (20× magnification) and quantification of periodic acid–Schiff staining in kidney of (B) male and (C) female mice using the Mantra 2 quantitative pathology imaging microscope. Data are expressed as mean ± SEM of 5–7 mice/group. A two‐way ANOVA was performed to detect differences.

NSD Pnpla3^I148M^ mice and female Pnpla3^I148M^ HSD mice showed no difference in triglyceride content in the kidney compared to controls (Figure 5A,B). However, triglyceride levels were significantly increased in male HSD Pnpla3^I148M^ mice as compared to controls (Figure 5A). This was in line with changes in renal morphology, where only male HSD Pnpla3^I148M^ mice exhibited an increase in renal injury (Figure 5C). All other Pnpla3^I148M^ groups showed a moderate trend for increased renal injury (Figure 5C,D).

### 3.4 Renal Serine–Threonine Kinases in Pnpla3^I148M^ Mice Are Altered in a Sex‐ and Diet‐Dependent Fashion

**FIGURE 6:**
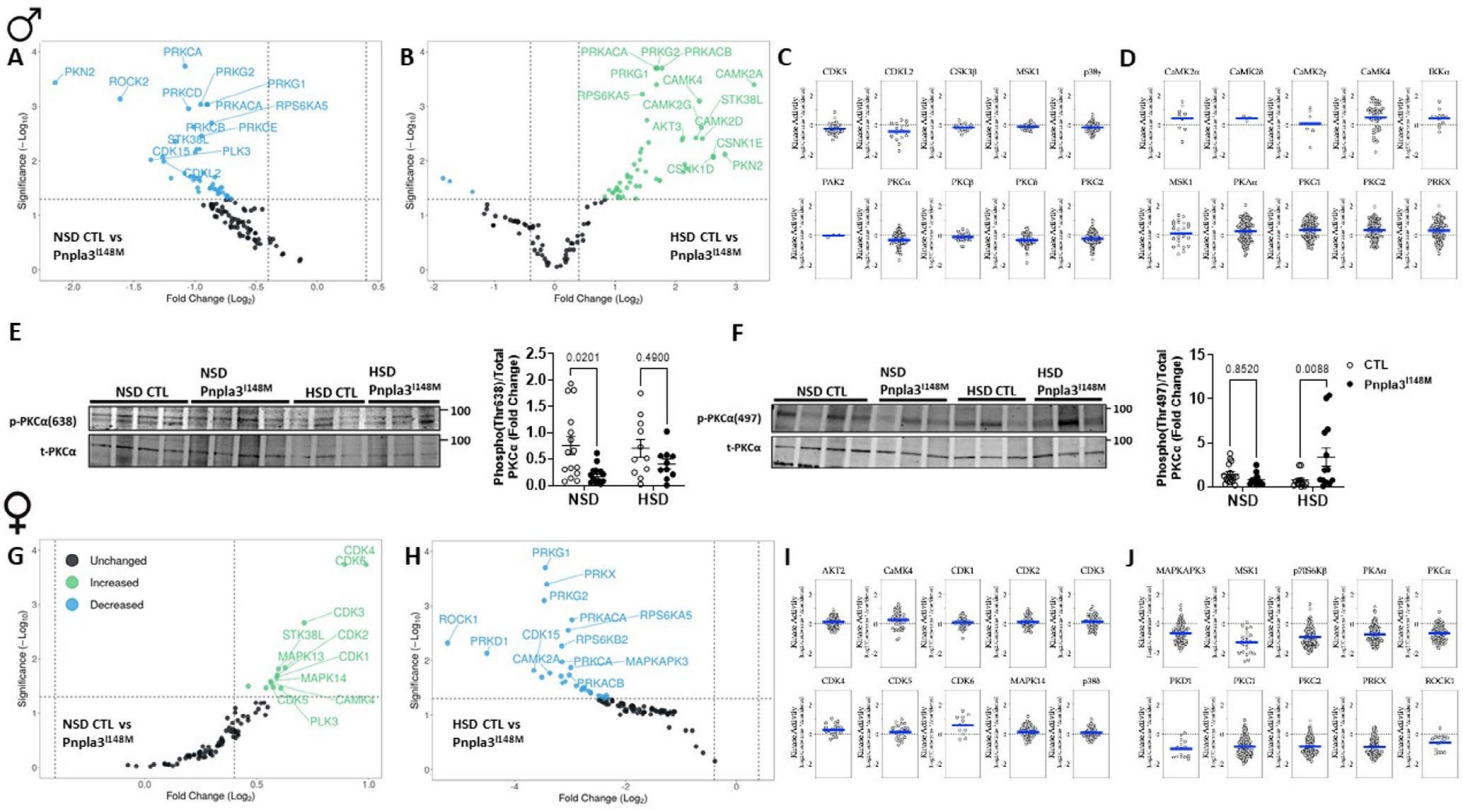
Pnpla3^I148M^ polymorphism alters serine–threonine kinases in a sex and diet‐dependent manner. Volcano plot of top 15 altered serine/threonine kinases (STK) in renal cortex of male (A, B) and female (G, H) Pnpla3^I148M^ mice compared to controls. Data are expressed as fold change compared to wild‐type (CTL) mice. *n* = 5/group. Quantification of STK by MEOW plots comparing male (C, D) and female (G, H) Pnpla3^I148M^ mice compared to control. The blue line represents the average of the activity for each subfamily of kinases (E). Representative western blot and quantification of phosphorylated PKCα (Th638) to total PKCα in male mice. (F) Representative western blot and quantification of phosphorylated PKCα (Th497) to total PKCα in male mice. Data are expressed as mean ± SEM of 10–12 mice/group. A two‐way ANOVA was performed to detect differences.

To investigate the potential mechanisms underlying renal hyperfiltration and injury, we performed a kinome analysis of renal cortices from control and Pnpla3^I148M^ mice fed either diet. Assessment of serine/threonine kinases (STK) in the renal cortex showed sex‐ and diet‐dependent effects of the Pnpla3^I148M^ polymorphism on the renal kinome (Figure 6A,B,G,H, Figure 3A–D). HSD male Pnpla3^I148M^ mice had the most kinases altered (55), followed by NSD males (45), HSD female Pnpla3^I148M^ (32), and NSD females (13). Altered kinases were predominantly in the CaMK and AGC kinase families (Figure S3A–D).

Upstream serine threonine kinase analysis showed that while male NSD Pnpla3^I148M^ mice showed mainly a decrease in kinase activity (Figure 6A), female NSD Pnpla3^I148M^ mice showed an increase in kinase activity (Figure 6G). This phenomenon was reversed in the HSD‐fed mice (Figure 6B,H). While female Pnpla3^I148M^ mice on NSD and HSD had very few shared altered kinases (2), male Pnpla3^I148M^ mice on NSD and HSD had more altered kinases in common (31) but in opposite directions. NSD male and female Pnpla3^I148M^ mice had few shared altered kinases (5). Male and female Pnpla3^I148M^ mice on NSD or HSD had similar kinase changes (8) but in opposite directions (Figure S3A–D).

Using the peacock plot analysis, we ascertained the differentially phosphorylated peptide substrates found in the experimental group (redline) targeted by a given kinase in relation to the number of randomly selected peptide substrates targeted by that same kinase in each of the thousands of random sampling events (gray histogram). A shift in the red line toward the right indicates higher confidence in the experimental group compared with the control and a change in kinase activity due to more differentially phosphorylated substrates than expected by random sampling (center of the histogram) for the given kinase (Figure S4A–D). This was recapitulated in further analysis using MEOW plots (Figure 6C,D,I,J).

The predominant kinases that showed a right‐ward shift and significant change in Pnpla3^I148M^ mice were protein kinase C alpha (PKCα), protein kinase A alpha (PKAα), and cGMP‐dependent protein kinase type 1 (PRKG1) and type 2 (PRKG2), which were altered in male and HSD female Pnpla3^I148M^ mice (Figure 6C,D,J). Isoforms of calcium/calmodulin‐dependent protein kinase 2 (CaMK2 α, ᵧ) were increased in HSD male Pnpla3^I148M^ mice as well as CaMK4 which was increased in HSD males and NSD females (Figure 6D,I). In NSD females, Cyclin‐dependent kinase 6 (CDK6) also had a right‐ward shift (Figure 6I). Peculiar to HSD‐fed mice, mitogen‐ and stress‐activated kinase 1 (MSK1 or RPS6KA5) and protein kinase, X‐linked (PRKX) had a right‐ward shift only in these groups, despite being a top hit in NSD males (Figure S4A–D, Figure 6D,J). We confirmed STK activity in male mice by Western blot analysis, detecting one of the highest hits (PKCα) and one of the lowest hits (GSK3β). Phosphorylated PKCα at Threonine 638, which is critical to prevent dephosphorylation and inactivation of PKCα [37] was decreased in NSD males while phosphorylated PKCα at Threonine 497, responsible for passive activation of PKCα [37, 38] was increased in HSD males (Figure 6E,F). Phosphorylated GSK3β at Serine 9, which leads to inactivation36, was also increased in NSD males but decreased in HSD males (Figure S4E).

Protein tyrosine kinase (PTK) activity was mainly decreased in Pnpla3^I148M^ mice, irrespective of diet (Figure S5A–D), with distinct kinase downregulation in each group.

## 4 Discussion

In the present study, we show for the first time that Pnpla3^I148M^ polymorphism promotes CKD incidence through renal hyperfiltration and hypertension, leading to cardiovascular dysfunction. These findings corroborate clinical evidence showing that Pnpla3^I148M^ carriers have reduced estimated GFR and higher CKD incidence independent of diabetes or MASLD [19, 20, 21]. The current data provide mechanistic insight, showing that the Pnpla3^I148M^ polymorphism causes sex‐specific alterations in the renal kinome and sodium transporter levels, leading to renal hyperfiltration, hypertension, and cardiovascular dysfunction that precede CKD development.

Increased GFR in Pnpla3^I148M^ mice was accompanied by elevated NHE3 and Na^+^/K^+^‐ATPase protein levels, suggesting that increased proximal tubule sodium reabsorption may activate tubuloglomerular feedback (TGF). Activation of TGF, drives afferent vasodilation to increase single‐nephron GFR [39]. Chronic hyperfiltration promotes glomerular hypertrophy, podocyte stress, and albuminuria, all key events in the onset and progression of CKD [40, 41]. In line with this, we found that HSD Pnpla3^I148M^ mice had increased albuminuria, and males, in particular, showed a trend toward morphological renal injury.

Renal hyperfiltration also elevates systemic blood pressure, increasing cardiac afterload and promoting cardiac remodeling [42, 43]. We found that Pnpla3^I148M^ mice are hypertensive, have reduced ejection fraction, and increased end‐systolic and end‐diastolic volume, irrespective of diet. The observed hypertension in the Pnpla3^I148M^ mice could be due to alterations in vascular or renal tubular function or a combination of the two. In the present study, indices of increased vascular stiffness were only observed in female Pnpla3^I148M^ mice. Given the kidney's role in long‐term regulation of arterial pressure, alterations in renal tubular transport may contribute to the development of hypertension in Pnpla3^I148M^ mice. However, it is possible that renal vascular resistance is increased in these mice as they age, which could contribute to a decline in GFR as well as protect against further renal glomerular injury. This hypothesis needs to be further examined in future studies.

Male Pnpla3^I148M^ mice have increased mitral valve early filling. This pattern reflects diastolic dysfunction and concentric remodeling typical of CKD‐induced cardiomyopathy. Interestingly, prior population studies found no direct association between Pnpla3^I148M^ and coronary artery disease [14], suggesting that cardiovascular dysfunction in Pnpla3^I148M^ carriers may arise secondary to renal pathology rather than atherosclerosis. Thus, the cardio‐renal axis is likely the primary pathway linking Pnpla3^I148M^ polymorphism to increased cardiovascular risk. We did not observe a change in collagen deposition in the heart between Pnpla3^I148M^ mice and controls at 24 weeks of age in this study. It is possible that, as the Pnpla3^I148M^ mice age, sustained hypertension will lead to increased cardiac collagen deposition and heart failure.

Renal kinome profiling revealed sex‐ and diet‐dependent alterations in PKCα, PKAα, PRKG1, PRKG2, CaMK4, and CaMK2. Inhibition of PKCα in Pnpla3^I148M^ mice (NSD males and HSD females) may be responsible for the increased levels of NHE3 observed in these mice, as previous studies have reported [44]. In HSD Pnpla3^I148M^ males, there was a trend for reduced NHE3 levels likely due to increases in PKCα and CaMK2, which are known to inhibit NHE3 [44, 45]. There was also a trend for increased α‐ENaC when compared to NSD control males. This likely contributed to the observed phenotype, as HSD Pnpla3^I148M^ males do not exhibit further increase in GFR at 22–24 weeks than what was observed in NSD Pnpla3^I148M^ mice. In NSD Pnpla3^I148M^ females, Na^+^/K^+^‐ATPase levels were increased in line with an increase in CaMK4 activity, which is known to enhance the levels and activity of Na^+^/K^+^‐ATPase via activation of the cAMP response element‐binding protein 1 (CREB1) [46, 47]. Hence, our findings suggest that sex‐dependent alterations in kinase activity in Pnpla3^I148M^ mice modulate sodium transporter levels, thereby leading to renal hyperfiltration. The mechanisms behind the sex differences in renal kinome activity observed in Pnpla3^I148M^ mice were not addressed in the present study, but could provide the basis for sex‐specific treatment of CKD progression in human patients with this polymorphism.

Several of the altered kinases exhibited in Pnpla3^I148M^ mice are known to affect kidney structure. For example, CaMK4 activation is known to accelerate progressive podocyte injury and loss [48]. Downregulation of cGMP‐dependent protein kinase (PRKG), type 1 and 2 (PRKG1, PRKG2) is known to increase kidney fibrosis and injury [49, 50]. This suggests that the Pnpla3^I148M^ polymorphism may induce a kinase signature consistent with early nephron injury. The identification of these altered kinases highlights the powerful potential of kinome analysis to identify additional targets for further exploration in future studies.

Sex differences were also observed in renal and cardiac phenotypes. Male Pnpla3^I148M^ mice exhibited higher blood pressure and albuminuria, while females showed reduced vascular compliance, particularly in HSD‐fed mice. This may be due to downregulation of PRKG1 and PRKG2 in HSD females, which are downstream targets and major mediators of the nitric oxide (NO/cGMP) signaling pathway [51]. Further experiments will be required to test this hypothesis.

Together, our findings provide mechanistic insight into clinical evidence showing that Pnpla3^I148M^ carriers exhibit accelerated eGFR decline and are at a higher risk of renal function loss, even in the absence of overt metabolic disease [19, 20, 21]. Our data demonstrate that the Pnpla3^I148M^ polymorphism may alter kinase signaling and proximal sodium reabsorption, triggering glomerular hyperfiltration, systemic hypertension, and cardiac dysfunction. The process is sexually dimorphic, with sex‐dependent kinase alterations that are responsible for pronounced hypertension and renal injury in males and greater vascular stiffness in females. Our results support the role of the Pnpla3^I148M^ polymorphism in the development of CKD; however, it remains to be demonstrated whether patients with this polymorphism also exhibit glomerular hyperfiltration before the development of CKD.

## Author Contributions

G.A. and D.E.S. conceived and designed the research. G.A., M.I.A., S.W., B.S.M., C.B.W., Z.A.K., T.D.H., and D.E.S. performed experiments. G.A, Z.A.K., and D.E.S. prepared figures. G.A. and D.E.S. drafted the manuscript. G.A., M.I.A., S.W., B.S.M., C.B.W., Z.A.K., T.D.H., and D.E.S. edited and revised the manuscript. All authors approved the final version of the manuscript.

## Funding

This work was supported by the National Institutes of Health, National Heart, Lung, and Blood Institute, 1R01HL174521‐01A1 (T.D.H. and D.E.S.), F31HL170972 (Z.A.K.), National Institute of General Medical Sciences of the National Institutes of Health, P30GM149404 (D.E.S.), National Institute of Diabetes and Digestive and Kidney Diseases, R01DK121797 (T.D.H.), National Institute on Drug Abuse, R01DA058933 (T.D.H.). The content is solely the authors' responsibility and does not necessarily represent the official views of the National Institutes of Health.

## Conflicts of Interest

The authors declare no conflicts of interest.

## Supporting information


**Figure S1:** Pnpla3^I148M^ mice show sex‐dependent alterations in vascular function. For assessment of vascular compliance, echocardiographic analysis by pulse‐wave Doppler imaging in B‐mode was done to determine abdominal aorta (AA) pulsatility index in (A) males (G) females. AA resistive Index in (B) males (H) females. AA peak systolic velocity in (C) males (I) females. AA Mean velocity in (D) males (J) females. Left common carotid artery (LCCA) pulsatility index in (E) males (K) females. LCCA resistive index in (F) males (L) females. Data are expressed as mean ± SEM of 10–15 mice/group. A two‐way ANOVA was performed to detect differences.
**Figure S2:** Pnpla3^I148M^ mice do not show increased cardiac collagen deposition. Representative image of picrosirius red staining of cardiac tissue for (A) males and (B) females, which were taken at 4× and 10× magnification using the color brightfield camera of the BioTek Lionheart FX automated microscope and the Mantra 2 quantitative pathology imaging microscope, respectively. 10× images were quantified using Image J software. Data are expressed as mean ± SEM of 5–7 mice/group. A two‐way ANOVA was performed to detect differences.
**Figure S3:** Pnpla3^I148M^ polymorphism alters serine–threonine kinases in a sex and diet dependent manner. (A–D) Kinome phyla of altered STK targets in renal cortex of male and female Pnpla3^I148M^ mice compared to CTL were derived by plotting the significant median final score (FC ≥ 1.30) and kinase statistic. Node color and size of the bubble plot on the paralogous phylogenic tree correspond to the final kinase score from the STK upstream kinase analysis. STK, serine–threonine kinase.
**Figure S4:** Further analysis of serine–threonine kinases alterations in Pnpla3^I148M^ mice. Quantification of STK by histogram peacock plots comparing male (A, B) and female (C, D) Pnpla3^I148M^ mice compared to control. (E) Representative Western blot and quantification of phosphorylated GSK3β (S9) to total GSK3β. Data are expressed as mean ± SEM of 10–12 mice/group. A two‐way ANOVA was performed to detect differences.
**Figure S5:** Protein‐tyrosine kinases are downregulated in Pnpla3^I148M^ mice. Volcano plot of altered protein‐tyrosine kinases (PTK) in renal cortex of male and female Pnpla3^I148M^ mice compared to controls. Data are expressed as fold change compared to wild‐type mice. *n* = 5/group.
**Table S1:** Cardiovascular parameters of male CTL and Pnpla3^I148M^ mice.
**Table S2:** Cardiovascular parameters of female CTL and Pnpla3^I148M^ mice.

## Data Availability

Raw data used for kinome analysis is available on Figshare (https://doi.org/10.6084/m9.figshare.32942291). All other data can be obtained from the corresponding author upon request.
