# Supplementary material for: The Human PNPLA3I148M Polymorphism Alters the Renal Kinome and Transporters to Cause Hyperfiltration and Hypertension
Source: FASEB Bioadv. 2026 Sep 19;8(9):e70146. doi: 10.1096/fba.2026-00222 (PMC13589268; doi:10.1096/fba.2026-00222)
Supplement: Supplementary file 1 [file FBA2-8-e70146-s001.docx]

**Supplementary Data**

**Figures and Figure Legends**

**Figure S1. Pnpla3^I148M^ mice show sex-dependent alterations in vascular function**

For assessment of vascular compliance, echocardiographic analysis by pulse-wave doppler imaging in B-mode was done to determine Abdominal Aorta (AA) Pulsatility Index in (A) Males (G) Females. AA Resistive Index in (B) Males (H) Females. AA Peak Systolic Velocity in (C) Males (I) Females. AA Mean Velocity in (D) Males (J) Females. Left Common Carotid Artery (LCCA) Pulsatility Index in (E) Males (K) Females. LCCA Resistive Index in (F) Males (L) Females Data are expressed as mean ± SEM of 10-15 mice/group. A two-way ANOVA was performed to detect differences.

**Figure S2. Pnpla3^I148M^ mice do not show increased cardiac collagen deposition**

Representative image of picrosirius red staining of cardiac tissue for (A) Males and (B) Females, which were taken at 4X and 10X magnification using the color brightfield camera of the BioTek Lionheart FX automated microscope and the Mantra 2™ Quantitative Pathology Imaging microscope, respectively. 10X images were quantified using Image J software. Data are expressed as mean ± SEM of 5-7 mice/group. A two-way ANOVA was performed to detect differences.

**Figure S3. Pnpla3^I148M^ polymorphism alters serine-threonine kinases in a sex and diet dependent manner**

(A-D) Kinome phyla of altered STK targets in renal cortex of male and female Pnpla3^I148M^ mice compared to CTL were derived by plotting the significant median final score (FC ≥ 1.30) and kinase statistic. Node color and size of the bubble plot on the paralogous phylogenic tree correspond to the final kinase score from the STK upstream kinase analysis. STK: serine-threonine kinase.

**Figure S4. Further analysis of serine-threonine kinases alterations in Pnpla3^I148M^ mice**

Quantification of STK by histogram peacock plots comparing male (A-B) and female (C-D) Pnpla3^I148M^ mice compared to control. (E) Representative Western blot and quantification of phosphorylated GSK3β (S9) to total GSK3β. Data are expressed as mean ± SEM of 10-12 mice/group. A two-way ANOVA was performed to detect differences.

**Figure S5. Protein-tyrosine kinases are downregulated in Pnpla3^I148M^ mice**

Volcano plot of altered protein-tyrosine kinases (PTK) in renal cortex of male and female Pnpla3^I148M^ mice compared to controls. Data are expressed as fold change compared to wild-type mice. n=5/group

**Table S1.** **Cardiovascular parameters of male CTL and Pnpla3^I148M^ mice**

|  | NSD CTL | NSD Pnpla3^I148M^ | HSD CTL | HSD Pnpla3^I148M^ |
| --- | --- | --- | --- | --- |
| Heart rate (bpm) | 464.9±7.7 | 443.2±7.3 | 428.3±12.2 | 431.0±15.1 |
| Heart weight (%body length) | 1.6±0.1 | 1.7±0.0 | 1.5±0.0 | 1.6±0.1 |
| Area; systole (mm^2^) | 14.4±0.7 | 16.2±0.4 | 13.1±1.0 | 14.9±0.9 |
| Area; diastole (mm^2^) | 20.9±0.7 | 22.2±0.6 | 19.5±1.3 | 19.5±1.3 |
| LVAW; systole (mm) | 1.4±0.0 | 1.2±0.0* | 1.3±0.1 | 1.2±0.0 |
| LVAW; diastole (mm) | 1.0±0.00 | 0.9±0.0 | 0.9±0.1 | 0.8±0.0 |
| LVPW; systole (mm) | 1.2±0.1 | 1.0±0.0 | 1.1±0.1 | 1.0±0.0 |
| LVPW; diastole (mm) | 0.9±0.5 | 0.8±0.0 | 0.8±0.0 | 0.7±0.0 |
| A’ (mm/s) | -32.1±3.1 | -24.8±1.7 | -19.3±1.9 | -19.1±1.7 |
| E’ (mm/s) | -17.6±1.5 | -19.7±1.8 | -19.7±1.4 | -18.6±1.2 |
| IVRT (ms) | 22.9±1.1 | 27.9±1.7 | 24.7±2.7 | 22.6±1.9 |
| MV E (mm/s) | 583.8±13.9 | 468.8±22.1* | 587.2±33.7 | 461.9±23.5* |
| A’/E’ | 2.0±0.2 | 1.5±0.2 | 0.9±0.0 | 1.1±0.11 |
| MV E/E’ | -32.7±2.57 | -25.5±2.0 | -27.4±1.9 | -24.3±1.8 |
| AA Mean Gradient (mmHg) | 0.2±0.0 | 0.1±0.0 | 0.2±0.0 | 0.2±0.0 |
| LCCA EDV (mm/s) | -43.6±7.3 | -37.2±6.3 | -23.2±3.6 | -28.5±3.6 |
| LCCA PSV (mm/s) | -151.2±19.9 | -124.5±10.1 | -115.1±10.1 | -126.8±11.5 |
| LCCA Mean Gradient (mmHg) | 0.03±0.0 | 0.03±0.0 | 0.02±0.0 | 0.02±0.0 |
| LCCA Mean Velocity (mm/s) | -87.4±11.7 | -76.2±7.2 | -64.3±5.6 | -69.9±5.0 |

LVAW= Left Ventricular Anterior Wall; LVPW= Left Ventricular Posterior Wall; IVRT= Isovolumic Relaxation Time; MV E= Mitral Valve Early Filling; AA=Abdominal Aorta; LCCA= Left Common Carotid Artery; EDV= End Diastolic Volume; PSV= Peak Systolic Velocity.

Data are expressed as mean ± SEM of 10-16 mice/group. A two-way ANOVA was performed to detect differences.

**Table S2.** **Cardiovascular parameters of female CTL and Pnpla3^I148M^ mice**

|  | NSD CTL | NSD Pnpla3^I148M^ | HSD CTL | HSD Pnpla3^I148M^ |
| --- | --- | --- | --- | --- |
| Heart rate (bpm) | 455.1±10.9 | 426.5±11.5 | 472.2±9.3 | 447.3±13.6 |
| Heart weight (%body length) | 1.3±0.1 | 1.5±0.0 | 1.2±0.0 | 1.3±0.0 |
| Area; systole (mm^2^) | 11.6±0.8 | 13.0±0.6 | 9.3±0.4 | 13.6±0.8* |
| Area; diastole (mm^2^) | 17.1±0.7 | 18.2±0.8 | 14.1±0.6 | 19.1±0.8* |
| LVAW; systole (mm) | 1.4±0.0 | 1.2±0.0* | 1.3±0.0 | 1.2±0.0 |
| LVAW; diastole (mm) | 0.9±0.0 | 0.8±0.0 | 0.9±0.0 | 0.8±0.0 |
| LVPW; systole (mm) | 1.2±0.0 | 1.1±0.1 | 1.1±0.0 | 1.0±0.0 |
| LVPW; diastole (mm) | 0.9±0.1 | 0.8±0.0 | 0.8±0.0 | 0.7±0.0 |
| A’ (mm/s) | -24.2±1.5 | -22.8±2.4 | -19.1±1.7 | -22.0±2.1 |
| E’ (mm/s) | -18.0±1.7 | -19.3±1.4 | -15.0±1.2 | -17.9±0.9 |
| IVRT (ms) | 24.3±1.3 | 27.9±1.7 | 20.9±1.4 | 22.8±2.2 |
| MV E (mm/s) | 487.3±31.4 | 473.0±28.1 | 488.7±41.3 | 451.5±18.5 |
| A’/E’ | 2.0±0.2 | 1.5±0.2 | 1.1±0.1 | 1.2±0.1 |
| MV E/E’ | -28.7±2.7 | -26.6±2.96 | -33.6±4.2 | -25.8±1.6 |
| AA Mean Gradient (mmHg) | 0.1±0.0 | 0.1±0.0 | 0.2±0.0 | 0.2±0.0 |
| LCCA EDV (mm/s) | -47.8±8.0 | -45.4±7.1 | -35.0±4.1 | -26.3±3.2 |
| LCCA PSV (mm/s) | -109.6±12.0 | -122.6±8.6 | -120.4±8.8 | -152.8±12.8 |
| LCCA Mean Gradient (mmHg) | 0.03±0.0 | 0.03±0.0 | 0.02±0.0 | 0.03±0.0 |
| LCCA Mean Velocity (mm/s) | -74.4±9.7 | -79.9±7.2 | -73.2±5.5 | -82.5±7.1 |

LVAW= Left Ventricular Anterior Wall; LVPW= Left Ventricular Posterior Wall; IVRT= Isovolumic Relaxation Time; MV E= Mitral Valve Early Filling; AA=Abdominal Aorta; LCCA= Left Common Carotid Artery; EDV= End Diastolic Volume; PSV= Peak Systolic Velocity.

Data are expressed as mean ± SEM of 8-16 mice/group. A two-way ANOVA was performed to detect differences.
